# Food in Migraine Management: Dietary Interventions in the Pathophysiology and Prevention of Headaches—A Narrative Review

**DOI:** 10.3390/nu17213471

**Published:** 2025-11-04

**Authors:** Tomasz Poboży, Kacper Janowski, Klaudia Michalak, Kamil Poboży, Julia Domańska-Poboża, Wojeciech Konarski, Iga Chuść

**Affiliations:** 1 Medicover Hospital, Aleja Rzeczypospolitej 5, 02-972 Warsaw, Poland; 2 Provincial Specialist Hospital in Ciechanów, ul. Powstańców Wielkopolskich 2, 06-400 Ciechanów, Poland; 3 Department of Neurosurgery, Brodnowski Masovian Hospital, 03-242 Warsaw, Poland; 4 Department of Rheumatology, National Institute of Geriatrics, Rheumatology and Rehabilitation, 02-637 Warsaw, Poland; 5 Medical Rehabilitation Center, Sobieskiego 47D, 05-120 Legionowo, Poland; 6 Faculty of Medicine, Medical Univeristy of Warsaw, 02-091 Warsaw, Poland; iga.chusc00@gmail.com

**Keywords:** migraine, diet, Mediterranean diet, ketogenic diet, omega-3 fatty acids, elimination diet, caffeine, alcohol, CGRP, gut–brain axis

## Abstract

**Background**: Migraine is a common, disabling neurological disorder with substantial genetic and environmental contributions. Dietary exposures are widely discussed by patients and clinicians as potential triggers or modifiers of attack frequency and severity. We synthesized contemporary evidence on dietary patterns, specific nutrients, and elimination strategies relevant to migraine prevention and management. **Methods**: We performed a narrative review of PubMed and Google Scholar (inception–August 2025) using combinations of “migraine”, “diet”, “nutrition”, “ketogenic”, “Mediterranean”, “omega-3”, and “gluten”. We prioritized randomized/controlled studies, recent systematic reviews/meta-analyses, and representative observational studies; evidence quality and applicability were appraised descriptively. **Results**: Higher adherence to a Mediterranean-style diet is associated with lower migraine frequency and disability in observational cohorts. Very low-calorie ketogenic diets significantly reduced monthly migraine attack frequency compared with isocaloric non-ketogenic comparators in an adult randomized controlled trial of participants with overweight or obesity (≥50% responder rate: 74% vs. 6%). Additional supportive evidence from uncontrolled studies, including those involving medium-chain triglyceride supplementation, further corroborates these findings. Omega-3 fatty acids (EPA/DHA) show prophylactic benefit in randomized trials and network meta-analyses, with favorable tolerability. Gluten-free diets may improve headaches in celiac disease and may help selected non-celiac patients. Alcohol (especially red wine) and high, irregular caffeine intake are frequently reported triggers, while evidence for specific foods/additives remains inconsistent. Weight loss and regular physical activity may further reduce burden in people with obesity. **Conclusions**: Current evidence supports recommending Mediterranean-style eating, consideration of omega-3 supplementation, and selective trials of ketogenic or elimination approaches in appropriate patients, alongside weight management and lifestyle optimization. High-quality, longer-duration RCTs using standardized dietary protocols and adherence biomarkers are needed to define dose–response relationships and enable personalized nutrition in migraine.

## 1. Introduction

Migraine is a complex neurological disorder characterized by recurrent attacks of moderate to severe head pain, typically affecting one side of the head, often unilateral, and accompanied by photophobia, phonophobia, and nausea. Research indicates that genetic elements significantly influence a person’s likelihood of experiencing migraines. Genetic susceptibility is substantial (heritability ≈50%), with contributions from both monogenic and polygenic mechanisms [1,2,3,4,5]. Its prevalence is reported to be up to three times higher in women than in men. While the exact reasons for this disparity remain unclear, several factors are thought to contribute to the difference (sex hormones, pregnancy effects, variations throughout the menstrual cycle, X-linked forms of migraines, and mitochondrial transmission) [6,7]. Migraine is a genuine problem and a frequent reason for disability and work absence due to complicated and recurring neurological incidents that can persist for hours or even days, profoundly affecting one’s life, everyday responsibilities, and job performance. The International Classification of Headache Disorders (ICHD-3) recognizes migraine with and without aura as the principal subtypes [6]. Migraine without aura is characterized by a headache with particular traits and related symptoms, whereas migraine with aura is highlighted by temporary neurological symptoms that frequently appear before or alongside the headache itself. Various theories exist that seek to clarify how migraine headaches develop. Understanding the relationships between blood vessels, neurons, and possibly mast cells is essential for uncovering the mechanisms that drive migraines. Contemporary pathophysiology highlights abnormal sensory processing and activation of the trigeminovascular system, with release of calcitonin gene-related peptide (CGRP) and other neuropeptides, together with neurovascular and inflammatory interactions [7,8]. Investigating how the brain interacts with diet and the gut microbiome in relation to migraines is an interesting subject for further research.

Diet is an important, modifiable element of lifestyle in which patients express strong interest. Determining food triggers might be difficult. Many types of diet, dairy products such as cheese and milk, as well as chocolate, citrus fruits, nuts, ice cream, tomatoes, onions, alcoholic beverages, coffee, caffeine, monosodium glutamate, histamine, tyramine, phenylethylamine, nitrites, aspartame, sucralose, and gluten have been considered in the literature as triggers influencing migraine headaches. Observational data and clinical studies suggest that dietary patterns, gut microbiota, specific nutrients, meal timing, and body weight may influence attack frequency, duration, and severity [9,10,11,12,13,14,15]. The identification of a number of dietary causes for migraines can lead to the development of strategies, mostly for better migraine maintenance and prevention. This narrative review summarizes recent evidence to inform pragmatic recommendations aligned with clinical practice and the neuroscience scope of this Special Issue.

## 2. Materials and Methods

This narrative review was conducted using a structured and transparent search strategy. PubMed was systematically searched from database inception to August 2025. Additionally, Google Scholar was screened manually to identify any relevant articles not captured in the database search. The search strategy combined the following terms: “migraine”, “headache”, “nutrition”, “diet”, “Mediterranean”, “ketogenic”, “omega-3”, “gluten”, “elimination diet”, “caffeine”, and “alcohol”. Only articles published in English were considered.

Inclusion criteria prioritized randomized controlled trials, controlled clinical studies, observational research (including cohort, case–control, and cross-sectional designs), as well as recent systematic reviews and meta-analyses. Eligible studies involved adult participants diagnosed with migraine and were published from database inception to August 2025. Research was included if it reported outcomes related to migraine or headache frequency, duration, intensity, or associated disability and quality-of-life measures. Exclusion criteria comprised non-peer-reviewed materials, including conference abstracts, theses, letters, editorials, and commentaries lacking original data. Studies were also excluded if they did not clearly define dietary exposures or migraine-related outcomes. In addition, research focusing exclusively on non-migraine headache disorders, such as tension-type headache or cluster headache, was omitted unless migraine-specific data were separately reported.

All included studies underwent descriptive quality appraisal to ensure robustness and relevance. Study quality was evaluated according to

- Study design (randomized controlled trial, controlled clinical trial, cohort, cross-sectional study, systematic review/meta-analysis);
- Adequacy of randomization and blinding where applicable;
- Sample size and population characteristics;
- Clarity and standardization of dietary intervention protocols;
- Assessment of diet adherence (self-report vs. objective biomarkers, where reported);
- Validity and consistency of migraine outcome measures (e.g., monthly migraine days, headache frequency/intensity, disability scores);
- Length of follow-up and completeness of outcome reporting;
- Reporting of withdrawals, adverse events, and tolerability.

Studies with stronger methodological rigor were prioritized in the interpretation. Backward citation tracking identified additional studies. Due to the heterogeneity of interventions/outcomes, no quantitative synthesis was conducted.

## 3. Results

### 3.1. Mediterranean-Style Dietary Pattern

**Figure 1:**
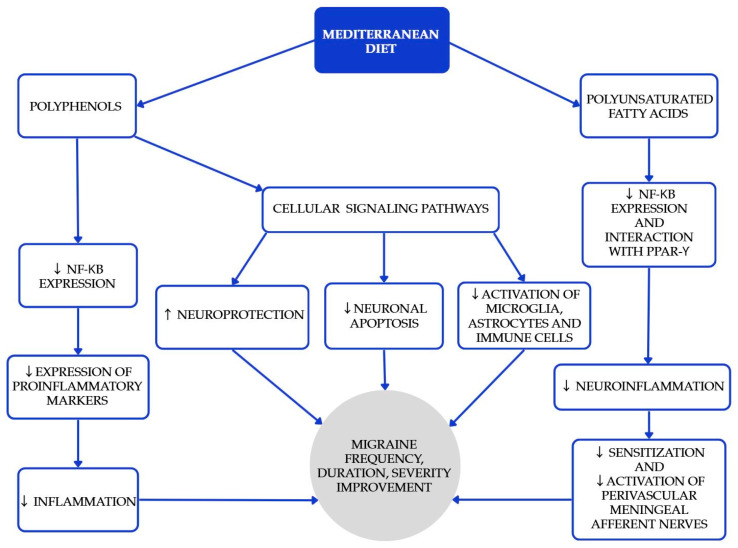
Proposed mechanisms underlying the beneficial effects of the Mediterranean diet and polyunsaturated fatty acids (PUFAs) in the prevention and attenuation of migraine symptoms. Blue elements indicate intermediary biological pathways involved in neuronal protection, inflammation reduction, and neuromodulation, while the gray element represents the overall clinical outcomes. Colors are used solely for visual organization and do not signify additional categorical meaning [21,22,23,24].

The Mediterranean diet (MedDiet) is one of the most researched and recognized dietary patterns globally, known for its numerous health benefits in cardiovascular diseases, diabetes, obesity, metabolic syndrome, carcinogenesis, cognitive function, and age-related diseases. Beyond the benefits mentioned above [16,17,18], higher adherence is associated with lower migraine frequency, shorter duration, and lower disability scores in cross-sectional analyses [19,20]. Polyphenols play a significant role in the MedDiet, primarily because of their antioxidant properties [18]. Furthermore, certain polyphenols act as bioactive nutripharmacological compounds that affect many biochemical processes. The MedDiet emphasizes low consumption of meat (especially red meat as beef, pork, and lamb), processed meats, butter, ice cream, and other whole-fat dairy products; abundant consumption of extra-virgin olive oil, vegetables, and fruits (processed as little as possible); nuts, legumes, cereals, fish, and shellfish, as great protein sources; and moderate amounts of red wine (one glass) [17]. Selected Mediterranean diet products and their recommended approximate intake values are shown in Table 1. The study conducted by Arman Arab et al. aimed to investigate the relationship between adherence to the Mediterranean dietary pattern and various features of migraine headaches, including their frequency, duration, and severity [19]. Questionnaires assessing dietary intakes, as well as measurement with MHIS (migraine headache index score) and HIT-6 (headache impact test) neurological scales, were used. The Mediterranean diet was associated with lower headache frequency, duration, and lower MHIS and HIT-6 score for those with higher adherence to the MedDiet compared to lower adherence. Bovenzi et al. found that low adherence to the MedDiet resulted in high-frequency episodic and chronic migraines in a group of 170 migraine patients [20]. Moreover, a significant negative correlation was observed between the HIT-6 scale and adherence to the MedDiet, which confirms that the MedDiet has a positive influence on migraine headache management and migraine chronification. While causality might be hard to infer, these results, together with biological plausibility, support recommending a MedDiet as a valuable pattern for patients with migraine. The mechanisms underlying the beneficial effects of the MedDiet (and PUFAs) are summarized in Figure 1.

**Table 1:** Selected Mediterranean diet products and their recommended approximate intake values. Bioactive compounds are indicated in parentheses [25,26,27,28,29,30,31].

Foods	Recommendations [26,31]
Fish (PUFAs) [25]	150 g of oily fish, two or three times a week
Olive oil (phytoalexins, oleuropein, oleocanthal, hydroxytyrosol) [27]	Every meal
Legumes (ferulic acid, genistein, kaempferol) [28]	Min. 2 serves weekly
Nuts (quercetin, resveratrol) [29]	1–2 serves daily
Red wine (resveratrol) [29,30]	In moderation/Occasionally (1 glass, 100–150 mL)
Apples, berries, grapes, persimmons, strawberries (fisetin) [29]	Fresh fruit: 1–2 serves daily Fresh vegetables: min. 2 serves daily
Carrots, tomatoes, peppers, lettuce, cucumber, spinach (luteolin) [29]	
Herbs (kaempferol) [29]	As a food additive/meal seasoning
Curcuma longa (curcumin) [29]	

### 3.2. Ketogenic Diet and Ketone Supplementation

**Figure 2:**
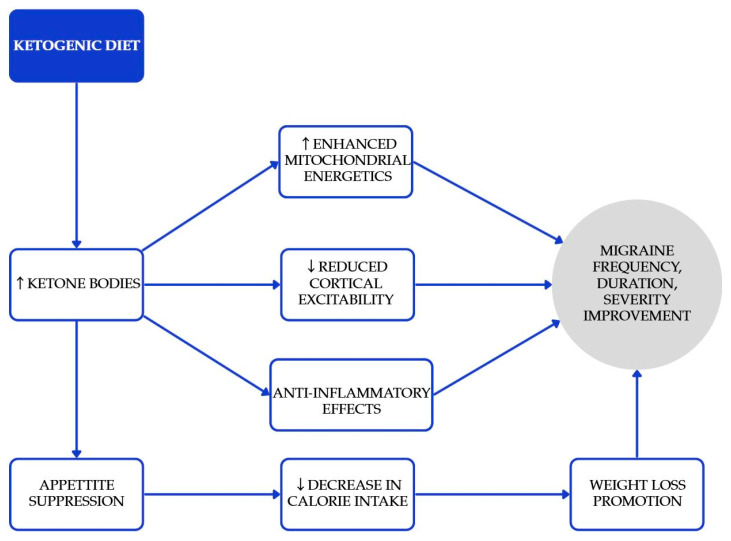
Proposed mechanisms underlying the beneficial effects of the ketogenic diet in the prevention and attenuation of migraine symptoms. Blue elements represent intermediate physiological processes associated with increased ketone body availability, while the gray element denotes the overall clinical outcomes. Colors are used solely for visual organization and do not indicate additional categorical meaning [32,33,36,39].

The ketogenic diet (KD) is characterized by low carbohydrate consumption and moderate restriction on protein, all designed to promote ketosis while allowing for unrestricted fat intake [32]. KD can elevate the levels of ketone bodies (KBs), which are intermediate products formed during the oxidation and breakdown of fats in the liver [33]. These ketone bodies have the direct effect of suppressing appetite and lowering calorie consumption [34]. Research has demonstrated that the ketogenic diet (KD) can effectively promote weight loss, reduce hyperinsulinemia, and enhance insulin sensitivity [35]. There are studies which claim ketone bodies have some beneficial effect on the prevention or relief of migraine attacks [36]. Proposed mechanisms relevant to migraine, including enhanced mitochondrial energetics, reduced cortical excitability, and anti-inflammatory effects, are summarized in Figure 2 [32,33,34,35,36]. In an RCT of adults with overweight/obesity and episodic migraine, a very low-calorie ketogenic diet (VLCKD; ~800 kcal/day) achieved a ≥50% responder rate of 74% versus 6% with an isocaloric non-ketogenic diet [37]. In one study, 14 individuals with episodic migraines participated in a one-month MCT supplementation, without making any other changes to their diet [38]. MCT supplementation, where approximately 60% of caloric intake derives from MCTs, primarily leads to energy production. However, when consumed in excess, acetyl-CoA can accumulate, which in turn stimulates the biosynthesis of ketones. By the conclusion of the study, patients reported a noticeable improvement in the frequency, duration, and severity of their migraine symptoms. Beneficial results with KD were achieved in more studies [39]. A three-month ketogenic diet (KD) has been associated with a reduction in painful symptoms for patients suffering from drug-resistant chronic migraines. The duration of migraine episodes has decreased from 24 h to 5.5 h. These improvements may indicate an enhanced quality of life for those individuals. KDs may be considered for motivated, appropriately supervised patients, particularly when weight loss is also indicated; monitoring for nutrient adequacy and tolerability is essential.

### 3.3. Elimination Approaches and Gluten-Free Diet

EDs target patient-specific triggers identified through structured diaries and planned re-challenge. In celiac disease, a gluten-free diet (GFD) substantially reduces headache prevalence and may achieve complete resolution in many patients [40,41]. In non-celiac patients, evidence is heterogeneous; a time-limited, structured GFD trial can be reasonable in those with compatible gastrointestinal symptoms or serological markers, ideally with dietitian support.

Elimination diets (EDs) remain the subject of many studies. EDs involve identifying specific dietary ingredients that might trigger undesirable reactions and subsequently removing them from a person’s diet [13]. Utilizing ketogenic diets can offer advantages, but it also comes with downsides. Benefits must be balanced against nutritional risks and potential for undue restriction. Caution is needed when implementing it, as complete elimination of certain food groups might result in nutritional deficiencies, potentially leading to various health issues, mental health disorders, or infections [42]. Consuming a particular food does not necessarily lead to a headache. It should be mentioned that the quantity of food and the timing of exposure can significantly affect the results. Additionally, there are instances where a noticeable delay occurs between eating a trigger food and the onset of a migraine, which makes it much more difficult to determine whether or not this food actually contributes to the exacerbation of a migraine attack. The role of dietary restriction in managing patients with migraines remains a contentious issue within the field of headache management [43]. For example, a gluten-free diet has shown remarkable effectiveness in patients with celiac disease, significantly reducing the frequency of headaches and migraines from 51.6% to a complete absence in some cases. This dietary approach not only helps manage headaches associated with celiac disease but also leads to total resolution in up to 75% of patients [40]. A gluten-free diet may help reduce intestinal inflammation and lower antibody levels, which could enhance nutrient absorption. Patients with celiac disease often show significant IgA reactivity with blood vessel structures in the brain, along with notable activation of the immune and inflammatory responses [41].

### 3.4. Omega-3 Polyunsaturated Fatty Acids

Omega-3 polyunsaturated fatty acids (PUFAs) exert positive effects on cardiovascular health, including atherosclerotic plaque morphology modification, LDL oxidation, cholesterol distribution, glucose homeostasis, and endothelial function. Higher levels of these fatty acids are linked to a reduced risk of developing cardiovascular disease [44]. Omega-3 PUFAs provide advantages for brain function and mental well-being thanks to their strong ability to reduce inflammation, alleviate pain, impact neurotransmitters, and fight oxidative stress. These properties help in controlling neuroinflammation, transmitting pain signals, improving mitochondrial function, and regulating mood [45,46]. Especially eicosapentaenoic acid (EPA) and docosahexaenoic acid (DHA) are essential for the nervous system and play a key role in several physiological functions [45]. It has been demonstrated that EPA and DHA have a positive impact through an anti-inflammatory mechanism, reducing nociceptive responses and inhibiting the dilation of blood vessels in patients suffering from migraines [24,47,48]. These properties could be advantageous in migraine prophylaxis or treatment. The meta-analysis conducted by Tseng et al. has demonstrated that high dosage of EPA and DHA leads to a reduction in both the number of migraines and their intensity (in contrast to placebo) in all the interventions examined [49]. A high-dose regimen, defined as eicosapentaenoic acid/docosahexaenoic acid (EPA/DHA) 180 mg/120 mg taken in six capsules per day, was associated with the greatest decrease in both migraine frequency and intensity among all evaluated prophylactic interventions. It should be mentioned that EPA/DHA supplementation was characterized by good acceptance and high adherence among patients. Standard medications frequently present greater rates of side effects and lower levels of compliance [24]. A combination of efficiency, safety, and acceptability showcases the promise of omega-3 PUFAs in providing a more holistic and patient-focused strategy for managing migraines.

### 3.5. Alcohol, Caffeine, and Food Additives

There are foods that are claimed to make migraine headache maintenance worse. Alcohol is considered as a trigger for migraine episodes due to an inflammatory, vasodilatory, and toxic physiological mechanism [50]. Alcoholic beverages were reported as a trigger by study participants with migraine. Wine, especially red, was recognized as the most common trigger among alcoholic beverages. Although moderate red wine intake (approximately one glass per day) is permitted within the Mediterranean diet due to its polyphenol content, alcohol is a well-recognized factor that may adversely influence migraine management. On average, patients reported that approximately 2.18 ± 1.3 standard glasses of red wine or 2.16 ± 1.9 glasses of vodka were sufficient to trigger a migraine attack [51]. Accordingly, the recommendation of non-alcoholic red wine—which preserves the antioxidant properties of its alcoholic counterpart—may represent a prudent and beneficial alternative for individuals with migraine. In a study of 684 patients, Mei-Ling Sharon Tai reported that coffee was the most frequently identified dietary trigger, followed by chocolate and foods rich in monosodium glutamate [52]. On the other hand, different studies observed no link between regular consumption of caffeinated drinks and the occurrence, length, or severity of headaches. Nowaczewska et al.’s study discusses the bidirectional properties of caffeine, which may contribute both to the progression of migraine headaches and to their treatment. The authors also highlight the occurrence of “withdrawal headaches” in individuals who consume caffeine daily and experience a sudden decrease in intake. According to the authors, individuals with migraine are advised to carefully monitor their caffeine consumption and limit their daily intake to no more than 200 mg. For those who choose to continue consuming caffeine, maintaining a stable and consistent daily intake is recommended to prevent withdrawal-related headache episodes [53]. As another factor that can provoke headaches, skipping meals is considered [54]. Other foods that have been reported in the literature to increase the frequency or severity of migraine headaches are summarized in Table 2.

**Table 2:** Foods considered as migraine triggers [45,46].

Vegetables	Onion
	Garlic
Dairy products	Milk
	Cheese and cheese products
Fruit	Oranges
	Strawberries
	Citric fruits
	Dry fruits, nuts
Western diet pattern	Cola, salted nuts, processed meat, fast food
Other food products and additives	Soft drinks
	Soy sauce
	Baked yeast foods
	Aspartame
	Carmosine

Evidence remains inconsistent and often derives from self-report rather than blinded provocation [13,14,15,55,56,57,58]. Clinicians should focus on individualized patterns rather than universal prohibitions.

### 3.6. Weight Management and Physical Activity

Some reports suggest that the frequency, occurrence, and severity of migraines seem to rise in correlation with body mass index. Obesity is associated with higher migraine frequency and chronification risk; behavioral weight loss may reduce attack frequency and disability [59,60,61]. Regular physical activity is beneficial for overall health and may modestly improve migraine burden, whereas low activity correlates with worse outcomes [62]. Dietary interventions should be integrated with weight management and exercise counseling when appropriate.

## 4. Additional Practical Recommendations for Clinicians

- Adopt a Mediterranean-style dietary pattern as the default foundation (high plants, whole grains, legumes, nuts, olive oil; moderate fish; low red/processed meat) [16,17,18,19,20].
- Discuss omega-3 intake: encourage oily fish twice weekly and consider EPA/DHA supplementation in prophylaxis, particularly when conventional preventives are poorly tolerated [24,49].
- Consider a time-limited ketogenic intervention (e.g., 8–12 weeks) for motivated patients—especially with overweight/obesity—under clinical supervision, with attention to nutrient adequacy and adherence [32,33,34,35,36,37,38,39].
- Use structured food/symptom diaries to identify personalized triggers; avoid broad, indefinite exclusions. If suspected, trial elimination with planned re-challenge; in celiac disease, institute a strict gluten-free diet [13,41].
- Counsel on caffeine regularity (avoid large fluctuations in the amount of caffeine consumed) and cautious alcohol intake; patients who identify red wine as a trigger should limit or avoid it [51,52].
- Integrate diet with weight management, sleep hygiene, stress reduction, and physical activity for comprehensive care [59,60,61,62].

## 5. Research Gaps and Future Directions

(1) Standardized endpoints and blinding: Trials should converge on core outcomes (monthly migraine days; ≥50% responder; disability indices), with predefined analysis plans and blinding strategies (e.g., sham foods, isocaloric comparators) to mitigate expectancy effects.
(2) Objective adherence biomarkers: Incorporate omega-3 index (erythrocyte EPA+DHA), β-hydroxybutyrate for ketosis verification, and dietary metabolite panels; link biomarker trajectories to clinical response.
(3) Dose–response and minimal effective dose: Define intake thresholds for EPA/DHA (e.g., g/day), carbohydrate ceilings for ketosis, and MedDiet adherence scores associated with clinically meaningful benefits.
(4) Phenotyping and precision nutrition: Stratify by aura status, sex, obesity/metabolic syndrome, sleep disorders, and comorbid mood/anxiety; test interaction effects and develop responder prediction models.
(5) Mechanistic readouts: Integrate CGRP levels, endothelial function (flow-mediated dilation), inflammatory lipid mediators, neuroimaging of cortical excitability, and the gut–brain axis (microbiome composition, SCFAs) to connect diet → mechanism → outcome.
(6) Long-term effectiveness and safety: ≥6–12-month interventions with maintenance phases, monitoring of nutrient adequacy (KD), lipid profiles, bone health, and quality of life.
(7) Real-world evidence and implementation: Pragmatic trials and registry-based studies comparing diet-first vs. standard care; cost-effectiveness and adherence facilitation (digital diaries, remote coaching).

## 6. Limitations

Heterogeneous interventions, variable dosing and adherence, reliance on self-report, and short follow-up durations limit certainty. Few trials incorporate adherence biomarkers or standardized outcomes, and blinded dietary provocation studies are rare. Generalizability across migraine subtypes and comorbidities remains uncertain.

The implementation of specific nutritional and dietary recommendations may require certain financial resources. Therefore, it is important to consider each patient’s individual economic capacity and to tailor the dietary and nutritional guidance accordingly. It is worth noting that establishing precise recommendations regarding the consumption of specific food products in defined quantities is challenging. Numerous factors influence the amount, quality, content, and activity of bioactive compounds in foods. These include, for example, the quality of breeding, cultivation or farming practices, methods of transportation, storage, product freshness, meal preparation, and the skills of the person preparing the food. Additionally, the bioavailability of nutrients may vary among patients due to individual factors such as comorbidities, overall health status, concomitant medications, dietary supplements, and interactions between them.

## 7. Discussion

**Figure 3:**
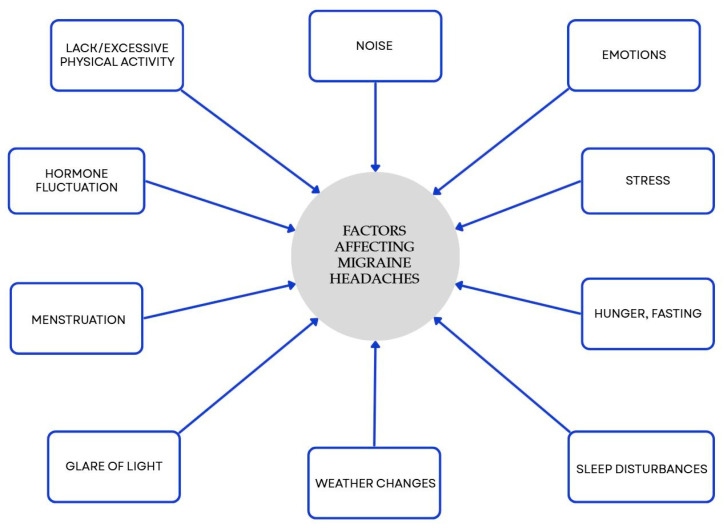
Factors influencing migraines beyond diet. Blue elements represent triggers that may promote migraine onset. Colors are used exclusively to improve visual clarity and do not convey additional categorical significance [50].

Many studies have shown a link between food choices and migraines. Nutrition is a central, modifiable component of lifestyle and valuable aspect of effective migraine management. It is worth considering strategies including a Mediterranean-style pattern, increased omega-3 intake, and—in selected patients—supervised ketogenic or targeted elimination approaches. Our results are consistent with previous clinical evidence supporting the role of dietary strategies in migraine man-agement (Supplementary Table S1). However, these relationships require validation through well-designed long-term studies. Some types of food, drinks, and components found in food can provoke headaches and/or migraines in people who are vulnerable to them. Several research efforts have shown that specific eating plans might modify the length, occurrence, intensity, and medication requirements in individuals suffering from migraines. Personalized, multidisciplinary care that integrates diet with lifestyle measures offers the greatest likelihood of reducing migraine frequency and associated disability. Diet is an important, but not the only, factor affecting migraine. There are many different factors, and some of them are presented in Figure 3.

There are no specific dietary recommendations for migraine headaches, but it is important to think about not just altering eating patterns but also lifestyle changes. It is hard to determine what quantities of meals can contribute to the triggering of a migraine and what bioactive substances in specific doses are responsible for their effects. A patient’s response to an eaten dietary trigger may depend on genetic factors and timing of exposure, which makes it even more difficult to interpret and establish a relationship between the consumption of a given food and the occurrence of a migraine. It is necessary to investigate and determine the content of individual substances in specific foods that can affect migraines, especially their absorption in the human body and bioavailability. It is worth keeping in mind the very likely interactions between foods consumed and the medications or dietary supplements used by patients, especially those not prescribed by healthcare staff and often taken without knowledge of where they come from. It is difficult to clearly distinguish in the studies whether a migraine was caused by a food, lifestyle, or mostly by other factors in the patient’s surroundings. Many factors and possibilities suggest that further research in the migraine area is necessary to establish standards and recommendations for migraine headache prevention, management, and treatment. All of the mentioned aspects should be considered when aiming for effective headache management.

### 7.1. Potential Contraindications and Patient Safety

Certain dietary interventions may not be suitable for all patients. For example, ketogenic diets should be used with caution or avoided in individuals with type 1 diabetes, advanced type 2 diabetes treated with insulin or SGLT2 inhibitors (due to risk of ketoacidosis), disorders of fatty acid oxidation, or hepatic and renal insufficiency [63]. Similarly, high-protein diets may be contraindicated in patients with chronic kidney disease, while strict low-fat regimens may be inappropriate in those with fat-soluble vitamin deficiencies or malabsorption syndromes [64,65]. Therefore, individualized assessment and medical supervision are essential before implementing any restrictive or specialized diet.

Safe implementation of dietary interventions requires regular clinical monitoring. Involvement of a professional dietitian is strongly recommended at the initiation and throughout the course of dietary therapy to ensure appropriate nutrient intake and to prevent deficiencies. Laboratory assessments should include metabolic panels, lipid profile, liver and kidney function tests, and, where appropriate, vitamin (e.g., B12, D) and mineral (e.g., iron, calcium, magnesium, potassium, sodium) levels. Frequency of monitoring should be individualized based on the patient’s condition, the restrictiveness of the diet, and comorbidities.

### 7.2. Feasibility and Adherence

The feasibility of dietary recommendations depends on patients’ socioeconomic conditions, culinary skills, and lifestyle. To improve adherence, dietary plans should be practical, culturally appropriate, and aligned with individual preferences and resources. Providing educational materials, behavioral support, and regular follow-up visits can enhance long-term compliance. Collaboration between physicians, dietitians, and patients is critical to ensure sustainable dietary changes and optimize clinical outcomes.

### 7.3. Future Research Directions

Future research should integrate genetic and microbiome profiling to advance precision nutrition in migraine management. Genetic variants influencing neuroinflammatory, metabolic, and vascular pathways—such as those related to CGRP signaling, mitochondrial function, and oxidative stress—may modulate dietary responsiveness [7,8]. Identifying genotype–diet interactions could enable stratification of patients and the development of personalized nutritional strategies.

In parallel, gut microbiota composition and function represent promising biomarkers and therapeutic targets [66]. Characterizing how dietary patterns, such as the Mediterranean or ketogenic diets, reshape microbial diversity and metabolite production (e.g., short-chain fatty acids, tryptophan derivatives) may clarify mechanistic links between diet and migraine pathophysiology.

Moreover, interactions between dietary interventions and pharmacological or nutraceutical treatments warrant systematic investigation. Many migraine patients concurrently use preventive or symptomatic medications as well as supplements. Understanding potential synergistic or antagonistic effects between diet-derived compounds and these agents is critical for optimizing therapeutic outcomes and minimizing adverse effects.

Future clinical trials should therefore incorporate genetic and microbiome biomarkers, evaluate interactions with standard pharmacotherapy and supplementation, and explore combination approaches that integrate nutrition with pharmacologic and behavioral strategies. Such precision-based frameworks may ultimately enable individualized, mechanism-driven dietary recommendations for migraine prevention and management.

## 8. Conclusions

Dietary interventions represent an important, modifiable component in migraine prevention and management. The Mediterranean diet is a well-studied approach known for its numerous health-promoting effects. Its implementation, in addition to better migraine management, may be associated with other health benefits. However, following it requires the patient’s willingness to cooperate and readiness to change daily habits.

The ketogenic diet shows promising results in improving migraine outcomes, likely through enhanced mitochondrial function, modulation of cortical excitability, and anti-inflammatory mechanisms. Prior to recommending a ketogenic diet, clinicians should ensure that the patient is fully informed about the potential risks and complications associated with this dietary approach. The ketogenic diet may represent an appropriate intervention for patients in whom good adherence can be anticipated.

Elimination diets may be appropriate for selected patients, particularly those with celiac disease or confirmed food sensitivities. However, empirical elimination without diagnostic confirmation can lead to unnecessary restriction and nutrient deficiencies. Due to the risk of malnutrition associated with elimination diets, appropriate patient education and careful monitoring of nutritional and health status should be ensured.

Supplementation with omega-3 polyunsaturated fatty acids (EPA and DHA) has shown beneficial effects in decreasing migraine frequency and intensity, suggesting a valuable adjunctive role in migraine prophylaxis. Supplementation with EPA and DHA appears to be a more feasible approach than modifying the entire dietary pattern; therefore, this strategy should be considered for patients in whom limited adherence to medical recommendations is anticipated.

Conversely, alcohol, excessive caffeine intake, and certain food additives may trigger migraine episodes in susceptible individuals, though current evidence remains inconsistent. Therefore, guidance on red wine intake should be tailored to each patient, and individuals should be informed about the levels of consumption regarded as appropriate within the Mediterranean diet. Clinicians should focus on identifying individual trigger patterns rather than recommending generalized dietary restrictions. It should be emphasized that these factors may adversely affect not only migraine management but also other aspects of overall health.

In summary, nutrition and lifestyle interventions should be regarded as part of a multidisciplinary, personalized approach to migraine management. Further well-designed, long-term studies are required to clarify causal mechanisms, establish standardized dietary recommendations, and optimize dietary–lifestyle interventions for migraine prevention and management.

## Data Availability

No new data were created or analyzed in this study. Data sharing is not applicable to this article.

## Supplementary Materials

The following supporting information can be downloaded at: https://www.mdpi.com/article/10.3390/nu17213471/s1, Table S1: Summary of Clinical Evidence on Dietary Interventions for Headache and Migraine Management.
