# Supplementary material for: Food in Migraine Management: Dietary Interventions in the Pathophysiology and Prevention of Headaches—A Narrative Review
Source: Nutrients. 2025 Nov 4;17(21):3471. doi: 10.3390/nu17213471 (PMC12609589; doi:10.3390/nu17213471)
Supplement: Supplementary file 1 [file nutrients-17-03471-s001.zip › nutrients-3922163-supplementary.pdf]

## Supplementary Table S1

| Author/Year | Design | Population | Intervention | Comparator | Duration | Primary Endpoint | Effect Size | Adherence/Biomarker | Safety/Notes |
|---|---|---|---|---|---|---|---|---|---|
| Di Lorenzo 2019 | RCT (cross-over) | Adults, N=35, overweight/obese, episodic migraine | VLCKD (800 kcal/day) | Isocaloric non-ketogenic | 8 weeks | ≥50% responder rate | 74% vs 6% | Ketosis verification (urinary/serum β-HB) | Generally well tolerated |
| Ramsden 2021 | RCT | Adults, N=182, chronic headache | High n-3, low n-6 diet | Control diet | 16 weeks | Headache days/month | -1.7 days vs control | Plasma fatty acids | Safe |
| Tseng 2024 | Network meta-analysis | RCTs pooled, N>2000 | High-dose omega-3 (EPA/DHA) | Placebo/other diets | Variable | Monthly migraine days | Superiority to placebo, some pharmacologics | N/A | Favorable tolerability |
| Zis 2018 | Systematic review/meta-analysis | Celiac disease with headache | Gluten-free diet | Normal diet | Variable | Headache prevalence | Marked reduction | N/A | Effective in celiac |

| | | | | | | | | | |
|---|---|---|---|---|---|---|---|---|---|
| Ameghino 2019 | Cohort | Celiac patients, N=188 | Gluten-free diet | None | Variable | Headache burden | Significant improvement | Dietary adherence questionnaires | Safe |
| Bongiovanni 2021 | Observational (refractory migraine) | Chronic migraine, N=23 | Ketogenic diet | None | 3 months | Attack frequency | ↓50% | Ketosis monitoring | Nutrient monitoring required |
| Wolkodoff 2020 | Pilot | Migraine patients, N=24 | MCT supplementation | None | 6 weeks | Attack frequency/intensity | Reduced burden | Dietary diaries | Safe |
| Bovenzi 2024 | Observational | Migraine patients, N=312 | Mediterranean diet adherence | Low adherence | Cross-sectional | Migraine chronification | Poor adherence → higher chronification risk | MedDiet scores | Observational only |
